# Medical Students and Professionals Facing the COVID-19 Pandemic: A Cross-Sectional Survey Study about Similarities and Differences

**DOI:** 10.3390/bs12060189

**Published:** 2022-06-15

**Authors:** Giacomo De Micheli, Giulia Marton, Davide Mazzoni, Laura Vergani

**Affiliations:** 1 Vita-Salute San Raffaele University, 20132 Milan, Italy; giaco.demi@gmail.com; 2 Department of Oncology and Hemato-Oncology, University of Milan, 20122 Milan, Italy; davide.mazzoni@unimi.it (D.M.); laura.vergani@ieo.it (L.V.)

**Keywords:** physicians, medical students, well-being, COVID-19

## Abstract

This study aimed at exploring the emotional reaction that medical students (MS) and professionals have faced during the COVID-19 pandemic and investigating the differences between the two groups. A total of 362 MS and 330 professionals filled in an online survey during the second outbreak of the COVID-19 pandemic in Italy. The outcome measures were psychological distress with the General Health Questionnaire, stress, fear for themselves, fear for family members and cohabitants, perceived control, anger, loneliness, and feeling abandoned by institutions with Visual Analog Scales (VAS) and two open-ended questions about their perceived difficulties and the perceived consequences of the pandemic. The results showed that the level of distress among the two groups was above the threshold (MS mean: 21.85; professionals mean: 21.25). The means of the VAS of MS and professionals showed different results for the two groups, and we analyzed them with independent samples *t*-tests and linear regressions. MS reported higher levels of perceived loneliness (*t*(673.177) = −1.970, *p* < 0.05), while physicians reported more fear for themselves (*t*(655.183) = 4.799, *p* < 0.001), anger (*t*(690) = 2.096, *p* < 0.05), and feeling abandoned by the institutions (*t*(690) = 7.296, *p* < 0.001). The open questions were analyzed considering the most frequent words used to describe their perceived difficulties and consequences; the specificity analysis emphasizes the differences in the words used by the two groups. In conclusion, MS and physicians reported similar levels of psychological symptoms. Physicians were mostly worried about themselves, they were afraid of getting infected, while MS suffered from loneliness and the missed possibilities concerning their education.

## 1. Introduction

The COVID-19 pandemic has been affecting people’s lives significantly for the past year [1] in many aspects, including mental health [2,3,4,5]. Many things that we have always taken for granted were taken away from us. We were separated from our loved ones, from our friends, and in some cases from our families [6]: we have been forced into our homes for months, making them the only place where we were feeling safe from the virus [7,8]. We have started to be afraid of hugs, and not feeling secure when spending time with other people. In addition to that, education as well has witnessed many challenges [9]. Medical education and work have been completely reorganized [10,11]: in the vast majority of the cases, online platforms replaced person-to-person activities, leaving things almost unaltered from the point of view of productivity but dramatically affecting the mental health of the people at home [12].

In many countries, medical students, independently from the year of attendance, started attending lectures [13] from home, even those that were supposed to be practical and would have implied a physical interaction with the environment nearby, not just a virtual one [14]. In particular, they had several limitations concerning their in-ward activities and laboratories, which are a crucial element for their professional formation [14,15]. The rationale for such reorganization of the teaching and learning activities was to prevent a drastic increase in the newly infected. However, several authors recently expressed important concerns about the potential negative impact that such limitations in the delivery of the didactic could have on the quality of students’ preparation [16,17]. 

In addition to the changes in the workload and the need to adapt to overcome the new situation, students all over the world mostly suffered from the pandemic’s emotional impact [18,19,20,21,22]. Medical students are already a population that suffers from anxiety, distress, and burnout, and the COVID-19 pandemic aggravated this condition [23]. For example, Indonesian medical students during the COVID-19 pandemic suffered from stress, anxiety, and depression [23]. Another example concerned Chinese medical students, one of the first countries to be affected by the pandemic. The students’ emotional reaction included anxiety and was related to various factors, including age and living conditions [22,24].

In addition to students, the daily activity of medical professionals has also been largely affected by the virus and its consequences. They have been putting themselves at risk every day carrying a tremendous weight on their shoulders when going back home to their families; moreover, since there was no visiting possibility, they were representing the bridges between patients and their loved ones, further increasing the pressure on themselves. This situation is having tremendous effects on healthcare professionals’ mental health and well-being, reporting high levels of psychological distress and high levels of fear, in particular for their families and patients [11]. In the Italian setting, healthcare workers were concerned for their patients and families, they felt alone, they reported high levels of anger, and they felt as if the situation was not under their control [11].

### Aim

In light of the above considerations, we wanted to compare a group of Italian medical students and a group of professionals in terms of emotional reactions and psychological symptoms related to the pandemic. Moreover, considering this complex and heterogeneous landscape, for each of the two groups, we wanted to identify the more frequently reported difficulties and perceived consequences caused by the pandemic.

## 2. Materials and Methods

### 2.1. Recruitment and Participants

This cross-sectional survey study [25] explored two different samples during the second outbreak of the COVID-19 pandemic in Italy. The two samples were enrolled with the Qualtrics platform through social media, mailing lists, and word of mouth starting from November 2020 until January 2021. The inclusion criteria were to be a medical student, currently enrolled in a university medical program, or a currently working physician, and they have to have been working/studying in Italy during the COVID-19 pandemic. Physicians and medical students that were not fluent in Italian were excluded from the study. 

Psychological symptoms were assessed through an Italian validated questionnaire, the General Health Questionnaire (GHQ-12; [26]). This instrument consists of 12 items, each one assessing the severity of a mental problem over the past few weeks using a 4-point Likert-type scale (from 0 to 3). The score was used to generate a total score ranging from 0 to 36. The presence of psychological symptoms is relevant if the answers are above the cut-off level of 13/14 [26,27].

Participants also answered to Visual Analog Scales (VAS) that were created ad hoc for this study. They explored several possible emotional reactions to the pandemic: stress, fear for themselves, fear for the family members and cohabitants, perceived control, anger, loneliness, and feeling abandoned by institutions. VAS use a line continuum, instead of the categories used by a Likert-type scale, to obtain more variability [28]. The scores ranged from 0 to 100 with high values indicating the worst condition; the VAS that investigated the perceived control, on the other hand, had high values indicating the best condition. 

In the final part of the questionnaire, participants were asked to answer two open-ended questions about their perceived difficulties and the perceived consequences of the pandemic.

### 2.2. Statistical Analysis

The analyses followed two main steps. In the first one, we compared medical students and professionals on psychological symptoms (i.e., GHQ-12) and emotional reactions (i.e., VAS) to the pandemic. We performed descriptive analysis, independent *t*-tests, and linear regression analyses (controlling for gender and region risk). These analyses were conducted using SPSS 26 [29] considering *p* ≤ 0.05 as statistical significance.

The answers to the open-ended questions about perceived difficulties and consequences were then analyzed through a lexicometry approach with the software T-LAB plus 2021 [30,31]. This software represents a set of linguistic and statistical tools for content analysis and text mining. More specifically, after the importation process (i.e., a series of processes that transform the texts into a set of tables integrated in the T-LAB database), T-LAB allowed to identify the most frequent words that were reported by the participants.

The T-LAB tool “specificity analysis” allowed us to check the “typical” and “exclusive” words that were used by students and professionals [32,33]. Exclusive words were identified through their presence in one group of answers only (e.g., used by students only or by professionals only). Typical words, defined by over-using in one of the two groups with respect to the other one, were identified by means of the chi-square test computation.

Finally, the T-LAB tool “thematic document classification” was used to obtain a representation of the corpus content through a few significant thematic clusters [32,33]. Each cluster consisted of a set of participants’ answers (i.e., documents) characterized by the same patterns of words. In this way, clusters were described through the lemmas and the variables (e.g., group of professionals vs. group of students) most characteristic of the texts by which they were composed. The degree of association between clusters and lemmas, and between clusters and levels of the variables (e.g, professionals vs. students) was described through the chi-square test [33].

## 3. Results

A total of 362 medical students and 330 professionals were enrolled. The mean age of medical students was 20.73 (SD = 1.97 min–max = 18–31), while the mean age of professionals was 47.88 (SD = 11.41 min–max = 26–73). The majority of the medical students were females (248; 68.5%), with 114 males (31.5%). Similar results were found in the physicians’ sample: 211 were females, (63.9%) with 116 males (35.2%) and 3 decided not to answer (0.9%). Restrictions in that period in Italy varied from region to region and were indicated with three different colors according to the regions risk scenarios established by the Italian Government [16]: “yellow areas”, with a moderate level of risk, “orange areas”, with a medium level of risk, and “red areas”, with a high level of risk. The majority of physicians, at the moment of answering our survey, were in the “yellow areas” (187; 56.7%), followed by “red areas” with 77 (23.3%), and only 66 (20%) were in the “orange areas”. Similar results were found for medical students: the majority were in the “yellow areas” with 195 (53.9%), followed by “orange areas” with 135 (37.3%) medical students, and only 32 (8.8%) were in the “red areas”.

We also asked participants if they had ever tested positive for COVID-19; most of the students answered no (329; 90.9%) as well as physicians (272; 82.4%). The same answer was given by most physicians (228; 69.1%) and medical students (184; 50.8%) when asked if they were ever quarantined.

The variables’ descriptive statistics can be found in Table 1. The normality of the distributions of all the VAS and GHQ-12 was acceptable (skewness ranging from −0.91 to 0.46; kurtosis ranging from −1.43 to 0.06).

In Table 1, we also report the independent samples *t*-tests that evaluated the differences between medical students and physicians. 

Table 2 presents the results of the linear regression models. Confirming the *t*-test results, physicians reported higher levels of fear for themselves and feeling abandoned by the institutions. No significant differences were found for the other variables.

### 3.1. The Words Used by Italian Medical Students and Professionals

During the importation stage, only the participants who responded to both the open-ended questions were considered. Each answer was considered as a document in the T-LAB environment. The final textual corpus thus consisted of 678 documents describing the perceived difficulties and 678 documents describing consequences. 

Table 3 presents the most frequent words used by the participants to describe their perceived difficulties and consequences.

Table 4, Table 5 and Table 6 illustrate the results of the specificity analysis. In detail, exclusive words (i.e., that appear in only one group) and the typical words (that appear significantly more often in only one of the two groups) are presented. This analysis emphasizes the differences in the words used by the two groups (medical students vs. professionals).

The exclusive and typical words that were used by the two groups to designate the difficulties highlight the different challenges that students and professionals faced during the pandemic. The main difficulties of medical students were related with staying at home and at the same time, maintaining the necessary motivation and concentration to study. On the other hand, professionals reported more difficulties related to the need to control and manage different aspects of their work, despite the presence of the COVID-19.

Concerning the perceived consequences, students appeared particularly worried about the possible negative outcomes on their careers (e.g., education, exams, etc.). On the other hand, professionals reported more often concerns related to the possibility, for themselves, for their relatives, and for their patients, of getting sick (Table 6).

### 3.2. The Perceived Difficulties

Through the tool’s thematic document classification, 323 answers to the question about the perceived difficulties were classified into 4 clusters. Table 7 presents the results of the thematic document classification of the participants’ answers about their perceived difficulties. Clusters were described through the words and the variables that mostly characterized a specific group (e.g., group of professionals vs. group of students).

Answers belonging to Cluster 1 “daily challenges” focused on the difficulties that participants faced during this period at a personal level. Examples of answers were “Staying all day at home” and “The feeling of alienation caused by staying all day at home, without seeing anyone other than your relatives. I miss living”.

Answers belonging to Cluster 2 “student’s life” focused on the lack of concentration and motivation, often related to the absence of social life. Examples of answers were “Losing much motivation to make the daytime productive, in absence of human contact” and “Concentration on the study/productivity/motivation in doing things”. This cluster was associated with the students’ group.

Answers belonging to Cluster 3 “people protection” focused on the respondents’ interest for the other people, both relatives and patients. An example of an answer was “Not being able to offer my professionalism to my patients, to patients affected by COVID-19, impotence in protecting relatives and dear people”. 

Answers belonging to Cluster 4 “management” focused on the difficulties in managing and controlling different critical aspects of the respondents’ lives. Examples of answers were “To manage activities when the routine no longer exists” and “To manage all the patients getting positive. In March and April [the difficulty was] being able to protect myself, considering the lack of protective equipment.” This cluster was associated with the professionals’ group.

### 3.3. The Consequences of the Pandemic

Through the tool’s thematic document classification, 448 answers to the question about consequences were classified into 4 clusters (Table 8).

Answers belonging to Cluster 1 focused on perceived “Consequences on education and practice”. An example of an answer was “I believe that the suspension of the training in the hospital will have negative consequences on the medical students’ education. From March to November, I did only 3 days of training (and some of my colleagues 0), while we expected three months. A significant part of our practical preparation is missing, and it will be difficult to recover”.

Answers belonging to Cluster 2 “Psycho-social consequences” focused on the possible impact of the pandemic at the psychological and social levels. Examples of answers were: “The impact on my mental health”, “Depression, the social and economic impact, that we are already seeing and there will be in the next few years”. 

Answers belonging to Cluster 3 “Socio-economic consequences” focused on the possible crash of the healthcare and economic systems. Examples of answers were “The insurmountable economic and healthcare crisis”, “A global economic crisis”, and “economic crisis, and distrust in the institutions”. 

Answers belonging to Cluster 4 “Consequences on family health” focused on the potential threat for the family members’ health. Examples of answers were “One or more dear people could suffer because of the pandemic” and “The risk of losing someone important”. This cluster was associated with the professionals’ group.

## 4. Discussion

Focusing on the comparison between medical students and professionals in the emotional reactions to the pandemic, we have identified some clear differences: the professionals reported higher levels of fear for themselves, and they felt more abandoned by the institutions than students. The result regarding the fear might be due to the age difference between the two groups: medical students were younger compared to the professionals, and, typically, the COVID-19 pandemic affects younger people with milder symptoms [34]. However, this result could be explained also considering the working situation of professionals that might have stayed in close contact with infected patients, in contrast to students who were forced to stay at home, in e-learning [13]. 

As regards the psychological symptoms, the level was above the threshold. In this case, the difference between the two groups was not statistically significant, meaning that the mental health of both groups was affected in the same way. This confirmed other studies’ results, finding a low level of mental health and a significant level of stress in the pandemic period, both in professionals [11] and in medical students [35,36]. In particular, these findings confirmed the ones retrieved in our previous study [37], concerning the psychological sequelae of the COVID-19 pandemic on mental health of medical and other healthcare students. In this particular study, while the psychological symptoms were above the threshold for both, some differences were found in the consequences and the difficulties faced. 

Students described their difficulties with words pertaining to academic life and lack of socialization. This confirms results from other studies [37,38]: for example, Cao and colleagues [22] found that the anxiety level of medical college students in China were positively related to worries about the influences of the pandemic on delays in academic activities and daily life. Professionals, instead, used lemmas linked to fatigue (“Fatigue”, “Impotence”, “To disconnect”), work (“Patient”, “Healthcare”, “Working”), and families (“Relative). These same themes were also reported by the physicians when writing about the consequences of the pandemic: specifically, they described the feared impacts of the pandemic on relatives and loved ones. These results were not unexpected during this pandemic period: other studies found that fear for family members was a common emotional reaction in professionals fighting against the COVID-19 pandemic [11,39], while compassion fatigue levels were high [40]. These results are in line with literature findings: healthcare providers faced many adversities such as limited resources, longer shifts, and an unaligned work–life balance [41]. These difficulties led to healthcare providers being afraid to infect their family members and deciding not to go home to protect their loved ones. They also felt emotional exhaustion [42]. This condition has lasted over time: authors found that in the first 18 months of the pandemic, many healthcare workers decided to leave their job [43].

Having considered the data previously exposed, we suggest that medical students were focused mainly on the consequences related to their education such as deterioration of the quality of teaching, delays in their clinical rotations, and practical classes. These results could also be considered from a wider point of view: the fatigue of the physicians in fact was confirmed by many studies [11,44] but the toxic consequences that psychological distress is bringing to medical students could imply that a new generation of physicians will suffer and, even more importantly, will not have the confidence that the medical practice requires and which is acquired with a proper medical education. 

### Limitations

This study has some limitations, mostly related to the convenient sample and cross-sectional design. As concerns the assessment of the emotional reactions to the pandemic, it was based on single ad-hoc measures, rather than validated scales with multiple items. As concerns the analysis of the open questions, the T-LAB software does not allow analyses with multiple predictors in the same model as in regressions (i.e., multiple statistical predictors of the typical words are not allowed). Finally, we did not explore the possible confounding factors related with the specific context (e.g., academic curricula, hospitals, departments, etc.) that the physicians were dealing with. Future research is encouraged to deepen this concept, exploring if fighting first in line versus working in less affected hospitals could impact the physicians’ distress. 

## 5. Conclusions

Our study suggests that an intervention to prevent the psychological distress of the future medical professionals would be useful. This pandemic has highlighted that the mental health of professionals is highly impacted by working conditions. For this reason, it has become essential to focus on the environment of the professionals to implement their mental health. As a future direction, institutions should take into consideration how to implement working conditions for professionals. In the same mind frame, the university institutions should tailor policies with the aim to consider a new and modified approach to learning also considering the mental health of the medical students. For example, the pandemic highlighted the need for university staff to be more in tune with the challenges that the students face by applying a more humanized approach.

## Figures and Tables

**Table 1:** Descriptive statistics and *t*-test results.

Variables	Medical Students’ Mean (SD)	Physicians’ Mean (SD)	*t*-Test		
			*t*	Degree of Freedom	*p*
Stress	65.42 (24.81)	64.77 (26.46)	−0.334	690	0.739
Fear for themselves	33.58 (26.59)	44.08 (30.58)	4.799	655.183	0.000
Fear for family members and cohabitants	72.62 (27.61)	72.31 (27.62)	−0.151	690	0.880
Perceived control	46.67 (27.16)	44.32 (26.08)	−1.161	690	0.246
Anger	46.74 (33.11)	52.02 (33.19)	2.096	690	0.036
Loneliness	51.35 (33.04)	46.21 (35.29)	−1.970	673.177	0.049
Abandoned by institutions	42.80 (33.77)	61.59 (33.91)	7.296	690	0.000
Psychological symptoms	21.85 (4.49)	21.25 (4.16)	−1.817	690	0.070

SD = Standard Deviation.

**Table 2:** Results of the linear regressions on each dependent variable.

	Stress	Fear for Themselves	Fear for Family Members and Cohabitants	Perceived Control	Anger	Loneliness	Abandoned by Institutions	Psychological Symptoms
Gender (1 = males)	β = −0.19 *p* < 0.001	β = −0.08 *p* = 0.025	β = −0.22 *p* < 0.001	β = 0.17 *p* < 0.001	β = −0.09 *p* = 0.014	β = −0.10 *p* = 0.006	β = −0.03 *p* = 0.404	β = −0.16 *p* < 0.001
Region risk (1 = orange)	β = 0.05 *p* = 0.200	β = 0.068 *p* = 0.085	β = −0.01 *p* = 0.837	β = −0.04 *p* = 0.264	β = −0.01 *p* = 0.783	β = 0.05 *p* = 0.187	β = −0.02 *p* = 0.587	β = 0.03 *p* = 0.375
Region risk (1 = red)	β = 0.07 *p* = 0.095	β = 0.10 *p* = 0.012	β = 0.04 *p* = 0.344	β = −0.04 *p* = 0.337	β = 0.05 *p* = 0.200	β = 0.04 *p* = 0.300	β = −0.04 *p* = 0.263	β = −0.04 *p* = 0.357
Occupation (1 = student)	β = 0.01 *p* = 0.842	β = −0.17 *p* = 0.012	β = 0.01 *p* = 0.867	β = 0.05 *p* = 0.195	β = −0.07 *p* = 0.068	β = 0.07 *p* = 0.091	β = −0.26 *p* < 0.001	β = 0.05 *p* = 0.212
R^2^	0.040	0.049	0.049	0.032	0.018	0.019	0.076	0.034
Adjusted R^2^	0.035	0.043	0.044	0.027	0.012	0.013	0.071	0.029
F (4684)	7.20 *p* < 0.001	8.82 *p* < 0.001	8.87 *p* < 0.001	5.71 *p* < 0.001	3.05 *p* = 0.017	3.34 *p* = 0.010	14.13 *p* < 0.001	6.08 *p* < 0.001

Notes. The dependent variables are listed in the first row. The names of the independent variables (predictors) are reported in the first column. Standardized regression coefficients (β) and significance (*p*) are reported.

**Table 3:** The most frequent words (and their occurrence) in the participants’ answers.

Difficulties	Consequences
Lack (42)	Relative (91)
Concentration (40)	Economic (68)
To be able (35)	To get sick (63)
Loneliness (34)	Dear (53)
Study (34)	Heath (47)
Social (32)	People (39)
To focus on (30)	Fear (37)
Patient (28)	COVID-19 (36)
Life (27)	Illness (32)
People (23)	Consequences (29)
To manage (23)	To lose (29)
Work (22)	Patient (27)
See (22)	Social (27)

**Table 4:** Exclusive words (and their occurrence).

Difficulties		Consequences	
Students	Professionals	Students	Professionals
Concentration (40)	Patient (28)	Economy (12)	Diagnostic (7)
Study (34)	To control (7)	Academic (11)	Delays (7)
To focus on (30)	Impotence (7)	Training (8)	National Health Service (5)
To study (14)	Safety (7)	Preparation (7)	Son (5)
Motivation (12)	Limitation (6)	Mental (7)	To manage (5)
Lesson (10)	Relative (5)	To find (6)	Children (4)
University (9)	Healthcare (5)	Practice (6)	
To stay (8)	Working (5)	Career (6)	
Daytime (7)	Distancing (5)	To go out (4)	
DAD (6)	To disconnect (4)	Practical (4)	
To lose (6)	Fatigue (4)	University (4)	
Productive (6)		Before (4)	
Academic (6)		To rescue (4)	
To live (6)		System (4)	
Exam (5)		To study (4)	
Contact (5)		Month (4)	
Focused (4)		Opportunity (4)	
Day (4)		Spending time (4)	
Stay (4)		Experience (4)	
Socialization (4)		Lesson (4)	
Monotony (4)		Lockdown (4)	

**Table 5:** Typical words used by the two groups to describe the perceived difficulties.

Students					Professionals				
	Occurrences in Students’ Answers	Total Occurrences	Chi-Square	*p*		Occurrences Professionals’ Answers	Total Occurrences	Chi-Square	*p*
Home	19	21	10.68	0.001	Work	19	22	15.77	<0.001
					Management	17	20	13.39	<0.001
					Uncertainty	14	17	9.91	0.001
					Future	9	11	6.20	0.012
					COVID-19	7	8	5.98	0.014
					To manage	16	23	5.88	0.015
					Freedom	10	13	5.53	0.018
					Lack	26	42	5.23	0.022

**Table 6:** Typical words used by the two groups to describe the consequences of the pandemic.

Students					Professionals				
	Occurrences in Students’ Answers	Total Occurences	Chi-Square	*p*		Occurrences in Professionals’ Answers	Total Occurrences	Chi-Square	*p*
Education	21	22	12.99	<0.001	Patient	24	27	24.52	<0.001
People	31	39	7.73	0.005	Infection	13	14	14.84	<0.001
To come back	12	13	6.41	0.011	Illness	24	32	14.39	<0.001
To lose	23	29	5.62	0.017	To get sick	40	63	12.17	<0.001
Impact	10	11	4.99	0.025	COVID-19	25	36	11.19	<0.001
Exam	9	10	4.28	0.038	Relative	53	91	10.16	0.001
To fear	18	23	4.02	0.045	Contagion	15	22	6.16	0.013
					Disease	6	7	5.45	0.019
					Contract	9	12	5.32	0.021
					Dead	10	14	4.94	0.026
					Isolation	8	11	4.22	0.039
					Increase	5	6	4.17	0.041
					Treatment	9	13	3.92	0.047

**Table 7:** Results of the thematic document classification on the perceived difficulties.

	Cluster 1 “Daily Challenges”	Cluster 2 “Student Life”	Cluster 3 “People Protection”	Cluster 4 “Management”
**Number of documents**	29 (8.98%)	114 (35.29%)	17 (5.26%)	163 (50.46%)
**Lemmas**	Day (28.91 *)	Concentration (24.26)	People (92.44)	To manage (18.41)
	Right (28.91)	Motivation (19.35)	Patient (26.82)	Management (14.45)
	To feel (28.91)	Human (19.35)	To protect (25.68)	Isolation (9.66)
	Possibility (28.53)	Social (17.44)	Dear (22.99)	Stress (9.66)
	To miss (21.57)	Life (12.93)	To see (9.65)	Activity (9.08)
	New (16.73	Daytime (11.56)		Missing (7.50)
	Balance (14.79)	Study (11.46)		Sociability (7.50)
	To face (10.72)	To lose (9.62)		Family (7.21)
	Home (6.05)	Lost (9.62)		Relative (6.90)
	To stay (5.36)	Productive (9.62)		Future (6.42)
	Fear (5.07)	Contact (9.62)		Distance (6.42)
		Distancing (7.69)		To control (6.42)
		Diverse (7.69)		Safety (6.42)
		Fact (7.69)		To be able (6.17)
		Absence (7.69)		Far (5.34)
		Lesson (7.62)		Lack (4.53)
		To study (7.29)		
		To find (7.29)		
Variables		Students’ group (21.09)		Professionals’ group (20.57)
		Younger participants (18.10)		Tested positive for COVID-19 (16.64)
				Older participants (8.69)

* Chi-square values are reported. All the values were significant at *p* ≤ 0.05.

**Table 8:** Results of the thematic document classification on the perceived consequences of the pandemic.

	Cluster 1 “Consequences on Education and Practice”	Cluster 2 “Psychological Consequences”	Cluster 3 “Socio-Economic Consequences”	Cluster 4 “Consequences on Family Health”
**Number of documents**	84 (18.75%)	57 (12.72%)	83 (18.53%)	224 (50%)
**Lemmas**	Treatment (53.08 *)	Impact (68.15)	Crisis (93.42)	Relative (47.14)
	Negative (28.45)	Psychologic (54.38)	Economic (78.08)	Dear (42.17)
	Hospital (28.45)	Doctor (43.24)	Healthcare (53.03)	People (25.27)
	Training (28.45)	Parent (37.43)	Collapse (39.68)	To infect (14.94)
	Education (25.18	Problems (36.12)	Friend (26.40)	To get sick (14.09)
	Impossibility (20.29)	Particular (30.84)	Increase (17.57)	Illness (11.50)
	Lack (20.29)	Mental (19.28)	Closing (17.57)	To transmit (8.24)
	Grandparent (20.29)	Return (18.16)	Level (17.57)	Time (7.75)
	Practice (20.29)	Future (15.73)	System (17.57)	Danger (7.35)
	Preparation (20.29)	Depression (13.00)	Crash (16.92)	Risk (7.21)
	I (16.22)	People (8.82)	General (16.70	Life (7.21)
	Month (16.22)	Social (8.40)	stress (10.25)	To lose (6.51)
	To recover (16.22)	Difficulties (7.70)	Pandemic (5.69)	To contract (6.20)
	Scarce (16.22	Economic (7.50)	Exam (4.00)	Dead (6.20)
	To stay (11.43)	Situation (5.67)	Consequence (3.96)	Job (6.19)
	Situation (10.45)		Normal (3.93)	Patient (6.08)
	COVID-19 (10.00)		To lead (3.93)	Loss (5.66)
	Academic (8.41)			Relative (5.44)
	Isolation (8.32)			Old (5.14)
	Possible (7.68)			Delicate (4.42)
	Period (7.68)			To put (4.42)
	Consequence (7.58)			To study (4.42)
				Health (3.99)
Variables			Female participants (7.28)	Professionals’ group (9.42)
				Older participants (7.61)

* Chi-square values are reported. All the values were significant at *p* ≤ 0.05.

## Data Availability

The data are available upon reasonable request.
